# Concrete vs. Abstract Processing in Repetitive Negative Thinking: Distinct Functional Effects on Emotional Reactivity and Attentional Control

**DOI:** 10.3389/fpsyg.2019.01372

**Published:** 2019-06-18

**Authors:** Monika Kornacka, Izabela Krejtz, Celine Douilliez

**Affiliations:** ^1^Faculty of Psychology, SWPS University of Social Sciences and Humanities, Warsaw, Poland; ^2^Univ. Lille, EA 4072 – PSITEC – Psychologie : Interactions Temps Émotions Cognition, Lille, France; ^3^Psychological Sciences Research Institute, University of Louvain, Louvain-la-Neuve, Belgium

**Keywords:** repetitive negative thinking, rumination, emotional reactivity, attentional control, attentional disengagement, dysphoria

## Abstract

Repetitive negative thinking (RNT) is a transdiagnostic process linked to emotional regulation impairment and involved in mood, anxiety, eating disorders and addictions. Attentional disengagement impairment is one of the factors hypothesized to be responsible for the recurrent and uncontrollable character of RNT. The aim of the present study was to empirically test this hypothesis with evaluation of disengagement from negative and RNT-related stimuli separately. Sixty participants were randomly allocated to one of three experimental conditions: abstract RNT, concrete RNT, and control condition (distraction). The change in their negative affect (PANAS) and their attentional disengagement impairment (exogenous cueing task) were measured. The analysis revealed that participants in abstract RNT condition presented higher emotional reactivity comparing to concrete or distraction conditions. The results indicated no differences between induction conditions in attentional disengagement. However, participants after concrete RNT induction had longer mean response times in exogenous cueing task comparing to control induction suggesting that they detected presented stimuli slower than participants in control condition. The results raised an important, from clinical point of view, question of distinctive impact of two types of RNT on emotional reactivity and attentional processes.

## Introduction

Research on Repetitive Negative Thinking (RNT) started with a focus on depressive rumination, that is “behaviors and thoughts that focus one’s attention on one’s depressive symptoms and on the implication of these symptoms” (Nolen-Hoeksema, 1991, p. 569). Depressive rumination was identified as an onset, maintenance and recurrence factor of depression (Nolen-Hoeksema, 1991; Watkins, 2008). Further research suggests that rumination is involved also in anxiety, eating disorders, posttraumatic stress disorder or addictions and might be classified as a transdiagnostic process (Ehring and Watkins, 2008; Watkins, 2008). This transdiagnostic perspective leaded researchers to consider rumination as a broader and content-independent process (RNT) linked to psychological disorders that are characterized by impaired emotional regulation. A meta-analysis confirms that rumination is a maladaptive emotional regulation strategy related to psychological disorders with the strongest effect size comparing to other emotional regulation strategies (e.g., avoidance, suppression, reappraisal) (Aldao et al., 2010).

Watkins (2004) suggests that maladaptive character of RNT depends on the mode of information processing and it does not depend on the content of negative thoughts *per se*. In RNT, there are two alternatives modes of information processing: abstract and concrete. Abstract RNT analyses causes, consequences and signification of an event. It refers to what was classically defined as rumination – repetitive and difficult to control dwelling on one or more negative issues (Ehring and Watkins, 2008). This type of RNT is characterized by a higher-order, more general processing of self-referent information and is often subject to cognitive distortions (e.g., generalization) (Ehring and Watkins, 2008; Watkins, 2008, 2015; Watkins and Nolen-Hoeksema, 2014). Abstract RNT is typically focused on reassessing the past in the search of general explanation or significance of a given event. For example, “what have I done to deserve that?”, “why it always happens to me?”. Contrary to reappraisal, abstract RNT is disconnected from the details of current situation and consequently does not lead to adaptive emotional regulation (Watkins, 2008). This kind of RNT is also focused on the discrepancy between one’s actual and ideal self and on the reasons for this discrepancy (Watkins and Nolen-Hoeksema, 2014; Watkins, 2015). Whereas, concrete RNT refers to attentional focalization on the present moment, one’s own emotional state and environmental details. The definition of concrete processing mode overlaps with mechanism of mindfulness meditation. However, it does not reflect the complexity of mindfulness construct as the non-judgemental attitude is not explicitly addressed in the concrete RNT (Watkins, 2015). Concrete RNT involves lower-order, non-conceptual and non-judgemental processing of present-moment experience (Watkins, 2008). According to the literature, abstract and concrete RNT have a distinctive impact on emotional reactivity, i.e., the changes in negative affect (Watkins et al., 2008). Abstract thinking is considered as maladaptive, while concrete thinking enhances emotional regulation (Moberly and Watkins, 2006; Watkins and Moulds, 2007; Watkins et al., 2008).

Recent literature suggests also that one of the factors responsible for the recurrence of maladaptive RNT is attentional disengagement impairment (Koster et al., 2005; Whitmer and Gotlib, 2013). However, there are only few studies testing this hypothesis and none of them took into account the processing mode theory (Donaldson et al., 2007; Morrison and O’Connor, 2008; Southworth et al., 2016) – an element that seems to be crucial for considering RNT in the perspective of emotional reactivity or emotional regulation. The distinctive impact of concrete and abstract RNT, to our knowledge, has never been explored in the context of attentional processes. In the present experimental study, we examine how abstract and concrete RNT affect emotional reactivity, i.e., the changes in negative affect measured pre and post RNT induction and attentional disengagement from negative and RNT-related stimuli. We describe our hypotheses after a brief review of the relevant literature on the RNT and its link to attentional processes.

### Theoretical Roots of RNT – The Actual-Ideal Self-Discrepancy and Abstract vs. Concrete RNT

A particular situation worth considering in the RNT perspective is the discrepancy between the actual and ideal self. According to Martin and Tesser (1989), this discrepancy between one’s actual situation and personal standards would activate RNT. Roberts et al. (2013) explored this prediction and the results of their study suggested that activating actual-ideal self-discrepancy increases significantly the level of state rumination. Additionally, this effect was moderated by trait RNT – high trait ruminators reported more state rumination in situation when actual-ideal self-discrepancy was induced comparing to low ruminators. In Roberts et al. (2013)’s study, state rumination was evaluated using the modified sustained attention to response task (SART, based on a go/no-go principle) with the hypothesis that state rumination would result in higher cognitive load and consequently in higher error rates. Surprisingly, the participants with activated actual-ideal self-discrepancy were more accurate, but slower during the SART comparing to control group. Roberts et al. (2013) impute those differences to the fact that RNT is more salient and has a greater emotional load. However, they suggest also that the results might be consistent with the hypothesis of impaired disengagement from the ruminative content in RNT. It is important to note that disengagement was not directly measured in their study. Moreover, Roberts et al. (2013) did not explore the differential impact of abstract and concrete RNT in the situation of actual-ideal self-discrepancy. Watkins, 2011 suggests that in this situation, abstract RNT may impair regulation of emotions by reducing attention to environmental details and to the present situation, increasing procrastination and rumination. On the contrary, concrete RNT would be adaptive and should reduce the emotional impact of a given situation. The differential effect of RNT on emotional reactivity after a failure induction—a prototypical situation of actual-ideal self-discrepancy—was supported in experimental studies. Participants using abstract RNT reported more negative affect comparing to those using concrete RNT (Moberly and Watkins, 2006; Watkins and Moulds, 2007; Watkins et al., 2008).

### Attentional Disengagement Impairment Hypothesis

Attentional deployment is one of the key elements of emotional regulation model (Gross, 2002). The literature suggests that attentional processes, particularly those linked with attentional focalisation on self-relevant stimuli and with self-immersion state might be associated with rumination (Treynor et al., 2003; Webb et al., 2012). Attentional processes in the RNT may be operated at two aforementioned processing modes (abstract vs. concrete RNT). According to Koster et al. (2011), difficulty to disengage attention is a key element increasing the risk of maladaptive RNT. RNT recurrence is affected by conflict identification and attentional control (Koster et al., 2011). An individual detecting a conflict between the actual and ideal self will be naturally driven to resolve this conflicting situation. There are two potential actions – resolving the problem by meeting the standards or, if the first is not possible, disengaging from the conflict, which requires reallocation of attentional resources (Carver and Scheier, 1998). An efficient disengagement enables reappraisal of the situation or the use of distraction – both enhancing an adaptive emotional regulation. In contrast, inefficient disengagement due to impaired attentional control will result in prolonged self-focus thoughts (i.e., RNT).

It is interesting to note that while Roberts et al. (2013) suggest that RNT might result in impaired disengagement, Koster et al., 2011 suggest that it is rather inefficient disengagement that is responsible for RNT. However, as further suggested by Koster et al., 2011, one might suppose that high ruminators are trapped in an impaired attentional control vicious circle where RNT becomes a habitual mode of thinking.

The literature on thoughts suppression might also enhance the explanation of the link between attentional disengagement and rumination (Wegner, 1994; Wenzlaff and Luxton, 2003). The attempts to suppress ruminative or focused on negative mood thinking might lead to changes in attentional processes causing the ironic effect of thoughts suppression and difficulties in disengaging from these repetitive negative thoughts (Wegner et al., 1987). Two mechanisms seem to be involved in the thought suppression process: monitoring of the occurrence of unwanted thoughts and distraction from those thoughts requiring more intentional control comparing to monitoring (Wegner, 1994). If an individual does not have sufficient mental control resources to perform an efficient distraction process, the attempts of control of negative or mood-related thoughts may lead to a paradoxical effect of difficulty in disengaging attention from rumination due to operating monitoring process (Wegner et al., 1993; Wegner, 1994; Wenzlaff and Luxton, 2003). Finally, Whitmer and Gotlib (2013) in their attentional scope model of rumination corroborate the hypothesis that RNT triggers attentional impairment (Roberts et al., 2013) and not the other way round. According to this model, RNT would cause a focalization on narrowed negative RNT-related content of thoughts. This restriction is reflected by an impaired attentional disengagement from RNT-related stimuli. The present study tests this hypothesis by experimentally inducing RNT and testing its impact on attentional disengagement from neutral, negative or RNT-related stimuli.

### Attentional Disengagement and RNT – Empirical Evidence

The impairment of attentional disengagement was previously explored mainly in dysphoric individuals (Koster et al., 2005). The number of studies exploring the link between RNT and attentional disengagement is limited. Recently three studies measured how trait RNT (i.e., the tendency to use rumination measured on self-report questionnaire) is linked to attentional disengagement (Grafton et al., 2016; Southworth et al., 2016; Vălenaş et al., 2017). Grafton et al. (2016) suggest that heightened ruminative disposition is associated with impaired attentional disengagement from negative information. Interestingly, they supported the effect previously noted in dysphoric participants (Koster et al., 2005) that the attentional disengagement impairment is visible only when controlled attentional processes are involved (i.e., the stimuli presentation time is around 1000 ms; Grafton et al., 2016). Southworth et al. (2016) supported those results using the same task. In their study, they not only evaluated trait disposition to use rumination, but also state RNT in response to negative event. The results suggest that both, state and trait rumination, are linked to impaired attentional disengagement. Vălenaş et al. (2017) further showed that attentional disengagement mediates the relation between rumination and exam anxiety. Moreover, in an experimental study, LeMoult et al. (2013) suggested that participants having more difficulties in disengaging their attention from negative facial expressions (on exogenous cueing task) reported also more RNT after a stress induction. However, this effect was only apparent among dysphoric individuals.

### The Present Study

The aim of the present study was to explore how rumination affects attentional disengagement and emotional reactivity (i.e., the changes in negative affect) by inducing experimentally two different processing modes of RNT (abstract vs. concrete) and measuring their impact on pre-post induction negative affect and on attentional disengagement from neutral, negative and RNT-related stimuli. We aimed at evaluating whether the impaired attentional disengagement is observed for negative stimuli in general, as it is suggested by the literature concerning dysphoria (Koster et al., 2005), or whether it is apparent only for RNT-related stimuli, as it might be inferred from the attentional scope model of rumination (Whitmer and Gotlib, 2013).

The present study offers three advantages over previous research. First, previous studies on the link between attentional biases and rumination used mainly tasks which measured general attentional bias to negative stimuli (dot-probe task; Koster et al., 2004), or attentional breadth (attentional breadth task; Grol et al., 2015), but do not clearly focued on attentional disengagement. For instance, Donaldson et al. (2007) showed that an induction of rumination (vs. distraction) did not affect general attentional biases measured in a dot-probe task. Using a similar design, Morrison and O’Connor (2008) failed to observe a significant difference between conditions (rumination vs. distraction induction) in pre-post measures of attentional biases (dot-probe task). The present study used exogenous cueing task (Koster et al., 2005) specifically created to evaluate attentional disengagement.

Second, previously described studies, directly measuring attentional disengagement (e.g., Southworth et al., 2016), used a cross-sectional design which prevented determining causal direction of the link between attentional disengagement and rumination. Most of the previous studies relied on self-reported measures of rumination. In the present study, rumination is experimentally induced using a goal cueing task, designed to induce rumination by activating actual-ideal self-discrepancy (Roberts et al., 2013). Finally, none of the previous studies explored the potential distinctive impact of the concrete vs. abstract RNT (Watkins, 2004) on attentional disengagement. Existing literature suggests that abstract RNT should increase emotional reactivity resulting in a higher level of negative affect after RNT induction comparing to concrete RNT or distraction (Watkins et al., 2008). According to previous studies (e.g., Roberts et al., 2013), abstract RNT should also impair attentional disengagement from negative stimuli.

## Materials and Methods

### Participants

Sixty participants were recruited at the campus of the University of Lille. The sample size for repeated measures ANOVA with within-between interaction was determinated (using GPower software) to detect medium sample size (0.25) with an alpha of 0.05 and power of 0.95. Two participants were excluded due to technical problems during one of the experimental tasks. Final sample consisted of 58 participants (34 females, *M*_age_ = 24.12, *SD* = 3.85), resulting in 20 participants in abstract RNT condition, 19 in concrete RNT, and 19 in the control condition. There were no significant differences between experimental groups in age (*F*(2,55) = 3.10, *p* < 0.1, η_p_^2^ = 0.10) and in gender (χ^2^ (2, *n* = 58) < 1).

### Materials

#### Goal Cueing Task (Roberts et al., 2013)

The task is designed to induce rumination by activating actual-ideal self-discrepancy. Participants were instructed to identify an ongoing and unresolved concern that had repeatedly came into their mind and caused them to feel negative or stressed during the previous week. Following the identification of the goal, in order to make the participants focus and dwell on that goal during ten minutes, they were asked to answer questions (e.g., “think about what was important about that difficulty in terms of your personal goals”) and rate the importance and recurrence of this goal on a 7-point Likert scale (e.g., “to which extent this unresolved goal has been bothering you at its worst”). Additionally, at the end of goal cueing task, participants were asked to provide six keywords that described their concern. Those personal keywords were further used in the attentional disengagement task (exogenous cueing task) as RNT-related stimuli.

#### RNT and Distraction Induction

The RNT induction, adapted from Watkins and Teasdale (2001), was used to induce abstract or concrete RNT and distraction. Participants were presented with a series of 15 sentences displayed on the screen, each for 40 s (see the sentences used in the task in the Appendix 1). The instruction differed depending on the experimental condition. In the abstract RNT condition, participants were instructed to focus on causes, consequences and signification, of each of the sentences (e.g., “*Analyze the causes, the consequences and signification of* the tension in your muscles,” “*Analyze the causes, the consequences and signification of* the way you react,” “*Analyze the causes, the consequences and signification of* how quick or slow your thinking is right now”). In the concrete RNT condition, participants were to focus their attention on the sentences (e.g., “*Focus your attention on* the tension in your muscles,” “*Focus your attention on* the way you react,” “*Focus your attention on* how quick or slow your thinking is right now”). In the distraction control condition, participants were asked to imagine a situation or an object and the sentence content was different comparing to abstract and concrete condition (e.g., “*Imagine* the shape of a large black umbrella,” “*Imagine* the layout of a typical classroom”). The full task lasted for 10 minutes in each experimental condition.

#### Exogenous Cueing Task

**FIGURE 1:**
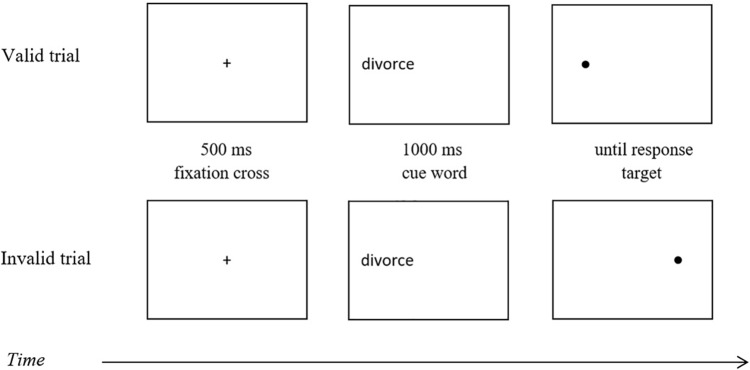
Schematic representation of valid and invalid trial in exogenous cueing task.

The task is designed to assess attentional disengagement (Koster et al., 2005). On every trial, a fixation cross is presented in the middle of the screen for 500 ms (see Figure 1). Next, a word cue appears for 1000 ms on the left or right-hand side of the screen. The target (a dot) is subsequently presented and remains on the screen until a response. Participants are instructed to indicate, as fast as possible, whether the target appeared on the right or left-hand side of the screen. They responded by pressing one of two keys (“d” for left target location and “k” for right target location) on a standard keyboard. In the valid trials, the target appears in the same location as the cue word, in invalid trials, on the opposite side. Cues consist of ten negative, ten neutral words and six RNT-relevant words. The neutral and negative cue words were selected on the basis of their affective valence and matched on familiarity and word length (the differences on frequency and length were non-significant between neutral and negative words) according to French Language Corpus (Interactive Language Toolbox; KU Leuven, 2014). RNT-related cue words were individually selected by each participant (see section “Goal Cueing Task”). The words were randomly selected from the set of each valence list. The task was preceded by 12 training trials, followed by 80 experimental trials with an equal number of valid and invalid trials.

Attentional disengagement was assessed by the attentional disengagement index proposed by Koster et al. (2005). The attentional disengagement index was calculated for negative stimuli (correct response time (CRT) to invalid trials with negative cue minus CRT to invalid trials with neutral cue). An analogous index was calculated for the RNT-related stimuli (CRT to invalid trials with RNT-related cue minus CRT to invalid trials with neutral cue). The positive value of attentional disengagement index indicates that participant had a longer disengagement time for emotional (negative or RNT-related) stimuli comparing to neutral ones, suggesting disengagement impairment. Negative or zero value of the index indicates no difficulty in attentional disengagement from negative or RNT-related stimuli.

#### Emotional Reactivity – Positive Affect Negative Affect Schedule

Participants’ emotional reactivity was assessed through changes in the Positive Affect Negative Affect Schedule (PANAS; Crawford and Henry, 2004; Gaudreau et al., 2006). The PANAS is a self-reported questionnaire that assesses positive and negative affect. The questionnaire is composed of 20 adjectives describing emotional states, ten for positive affect (e.g., excited) and ten for negative affect (e.g., distressed). Participants indicated on a 7-point Likert scale the extent to which the affect-related adjective described their current emotional state. The full version of PANAS was administrated four times, at the baseline and after each task in the procedure. In the present study negative affect subscale used in the further analysis had a good internal consistency in all four measure times (α = 0.88–0.93).

#### Trait Abstract RNT – Mini Cambridge Exeter Repetitive Thinking Style Questionnaire

The Mini-CERTS is a brief self-reported questionnaire assessing the processing mode of RNT (Douilliez et al., 2014). Abstract repetitive thinking was assessed using nine item subscale (e.g., “My thinking tends to get stuck in a rut, involving only a few themes”), each item is rated on a 4-point Likert scale. Abstract repetitive thinking subscale used in the present study has satisfying internal consistency Cronbach’s α = 0.70.

#### Ruminative Response Scale – Revised (RRS-R; Treynor et al., 2003)

The 10-item version of a self-reported questionnaire assessing rumination was used. The short version is composed of five items evaluating brooding rumination dimension (e.g., “Think about how alone you feel”) and five items evaluating reflection dimension of rumination (e.g., “Go away by yourself and think about why you feel this way”). The internal consistency of total scale was quite low Cronbach’s α = 0.62, the consistency of brooding was also relatively low (α = 0.64).

#### Beck Depression Inventory (BDI-II; Beck et al., 1996)

The BDI-II is a 21-item questionnaire assessing the presence and severity of depressive symptoms over the previous 2 weeks. Responses range from 0 (e.g., “I do not feel like a failure”) to 3 (e.g., “I feel I am a total failure as a person”). In the present study BDI-II had a good internal consistency with Cronbach’s α = 0.88.

### Procedure

Participants were recruited on the campus by the experimenter. During the testing phase, participants were alone in the experiment room. They were instructed to solicit experimenter’s help, if needed. All the tasks (including questionnaires) were programmed in Inquisit version 4 software and displayed on a 13.3 inches screen. All participants signed an informed consent form prior to their participation.

First, participants were asked to provide demographic information: age, sex, and educational level. Then, they filled a pre-induction PANAS (PANAS 1) to assess their emotional state. The PANAS was also administrated after each of the tasks presented below (PANAS 2 – after goal cueing task; PANAS 3 – after abstract vs. concrete RNT or control induction; PANAS 4 – after exogenous cueing task. The time 4 measures were not included in the analyses as they were added for the ethical reasons only in order to check whether participants’ emotional state after the end of experiment was reinstated at a similar level as the baseline).

Next, participants completed the goal cueing task in order to activate RNT process. Subsequently, participants were randomly assigned to one of the three conditions, two RNT (i.e., abstract vs. concrete) induction conditions and one control condition (distraction). Finally, participants completed the attentional cueing task in order to assess their attentional disengagement. Finally, they were asked to fill in the Mini-CERTS, RRS-R, and BDI-II. Participants were debriefed once the study was concluded. The study, including information phase, signing a consent form and debriefing, lasted between 50 and 60 min. Participants did not receive any incentives for their participation. The whole experimental procedure was approved by Institutional Behavioral Sciences Ethics Review Committee of the University of Lille (number of the approval: 2014-3-S23) and was carried out in accordance with The Code of Ethics of the World Medical Association (Declaration of Helsinki) for experiments involving humans.

### Statistical Analyses

First, to test the hypothesis of the differential impact of abstract vs. concrete on emotional reactivity, we computed a mix design ANOVA 3 (Experimental condition: abstract, concrete, distraction) × 2 (Time: Time 2, pre- RNT induction; Time 3, post-RNT induction) on the negative affect score from PANAS. Second, in order to test the impact of experimental induction of RNT on attentional disengagement, we run a mixed design ANCOVA 2 (Word type: negative, RNT related) x 3 (Experimental condition: abstract, concrete, distraction) on the attentional disengagement index with negative affect measured after RNT induction as a covariate. Finally, to assess the moderated effect of trait variables, we computed moderation models using conditional process models (Hayes, 2015). The relatively small sample size is one of the limitations of the present study. To address this limitation in moderating effect analyses, we chose to compute the moderator effect with the bootstrap method (with 5000 bootstraps), that is more suitable for the small samples (Hayes, 2015).

## Results

### Data Preparation

Erroneous responses (1.36%) on the attentional cueing task were excluded from statistical analyses. Those responses were identified following Koster et al. (2005) procedure for cleaning the data in exogenous cueing task. Reaction times shorter than 150 ms or longer than 1500 ms and RTs that deviating more than three *SD*s from the individual mean latency were also excluded from the data set used in the further analysis (5.45%).

### Statistics and Mean Comparisons Between Abstract RNT, Concrete RNT and Control Group

Mean and standard deviations by condition for all variables are presented in the Table 1. One-way ANOVAs were computed for each of the variables in order to assess the group differences between the RNT conditions (abstract RNT, concrete RNT and distraction). There were no significant differences in trait measures of rumination or depressive symptomatology across the three induction conditions.

**Table 1:** Mean and Standard Deviations for each variable by condition.

	Type of induction			Group comparison	
Variable	Abstract (SD)	Concrete (SD)	Distraction (SD)	F_(2,55)_	η_p_^2^
**Invalid RT**					
Neutral	371.74 (78.03)	433.91 (187.91)	341.07 (55.39)	2.80^2,+^	0.09
Negative	364.11 (73.69)	**434.49 (175.69)**	332.58 (56.59)	4.04^1,2,∗^	0.13
RNT-related	373.60 (68.33)	**443.02 (183.78)**	339.31 (63.09)	4.06^1,2,∗^	0.12
**Valid RT**					
Neutral	382.87 (77.98)	437.93 (150.81)	361.01 (50.27)	2.88^+^	0.09
Negative	383.84 (68.83)	442.28 (182.33)	359.12 (65.26)	2.49^+^	0.07
RNT-related	387.49 (62.43)	440.78 (161.29)	364.85 (52.54)	2.62^2,+^	0.09
**Cue validity**					
Neutral	−11.13 (39.72)	−4.01 (49.81)	−19.93 (35.65)	0.68	0.02
Negative	−19.72 (40.91)	−7.78 (63.85)	−26.54 (44.38)	0.67	0.02
RNT-related	−13.88 (35.29)	2.24 (47.03)	−25.53 (23.23)	2.47^+^	0.08
**Attentional disengagement**					
Negative	−7.63 (33.17)	0.58 (33.81)	−8.49 (24.19)	0.56	0.02
RNT-related	1.86 (36.84)	9.11 (33.56)	−1.76 (25.33)	0.51	0.02
Mini-CERTS AAT	23.00 (4.12)	24.31 (4.00)	24.58 (4.02)	0.83	0.02
RRS	23.68 (4.73)	23.95 (4.35)	24.00 (4.79)	0.02	<0.01
Brooding	11.89 (2.75)	12.47 (2.78)	12.68 (3.51)	0.34	<0.01
BDI-II	16.31 (8.63)	15.05 (10.80)	14.89 (9.15)	0.15^2^	<0.01
Personal words valence	2.60 (0.80)	2.55 (1.01)	2.27 (0.74)	0.83	0.03

*^∗^p < 0.05; ^+^p < 0.10. RT, response time; Mini-CERTS AAT, abstract analytic thinking from Mini Cambridge Exeter Repetitive Thinking Style Questionnaire; RRS, ruminative response scale; BDI-II, beck depression inventory –II. ^1^Bolded values are significantly different from the other means in a row according to post hoc analysis with Bonferroni correction. ^2^The ANOVA was computed on a transformed variable (logarithmic transformation) in order to improve the homogeneity of variance (for reaction times) or normality of distribution (for BDI-II).*

### Abstract vs. Concrete RNT Effect on Emotional Reactivity

**FIGURE 2:**
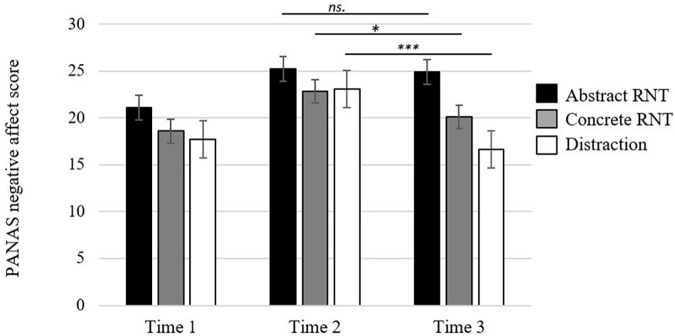
Negative affect score (PANAS) as a function of measure time and experimental condition. *^∗^p* < 0.05; ^∗∗∗^*p* < 0.001, *ns.* non-significant. Time 1 (baseline), Time 2 (after rumination induction – goal cueing task), Time 3 (after RNT mode induction).

First, a mixed design ANOVA 3 (Experimental condition: abstract, concrete, distraction) × 2 (Time: before vs. after rumination induction) was computed on negative affect subscale of PANAS in order to assess emotional reaction to RNT activation in goal cueing task and whether this change is different across experimental groups. The ANOVA revealed a significant effect of time (*F*_(1,57)_ = 40.02, *p* < 0.001, η_p_^2^ = 0.41), suggesting that participants’ negative affect increased after rumination induction (see Figure 2: Time 1 and Time 2). As expected, the interaction effect between Experimental condition and Time was not significant as at this stage of experiment all groups underwent exactly the same procedure of RNT activation (*Fs* < 1).

To test the direct effect of abstract vs. concrete RNT and distraction induction on emotional reactivity, a mixed design ANOVA 3 (Experimental condition: abstract, concrete, distraction) x 2 (Time: Time 2, pre-RNT induction; Time 3, post-RNT induction) was computed. The effect of Time, *F*_(1,53)_ = 24.40, *p* < 0.001, η_p_^2^ = 0.31, the main effect of Experimental condition, *F*_(2,55)_ = 3.37, *p* < 0.04, η_p_^2^ = 0.11, and Experimental condition x Time interaction effect, *F*_(2,53)_ = 7.81, *p* = 0.001, η_p_^2^ = 0.22, were significant. In line with expectations, *post hoc* comparisons with Bonferroni correction revealed a significant decrease of negative affect from pre-RNT induction to post-RNT induction for concrete RNT condition (*p* = 0.02; *M* = 22.84, *SD* = 7.52; *M* = 20.10, *SD* = 7.16, respectively), and distraction (*p* < 0.001; *M* = 23.05, *SD* = 8.05; *M* = 16.63, *SD* = 4.71, respectively). This difference was not significant for abstract RNT condition (*p* = 0.78; *M* = *2*5.20, *SD* = 6.38; *M* = 24.90, *SD* = 6.90, respectively), see Figure 2: Time 2 (after rumination induction) and Time 3 (after RNT processing mode induction).

### RNT Induction Effect on Attentional Disengagement

We run analysis of covariance, a mixed design ANCOVA 2 (Word type: negative, RNT-related) × 3 (Experimental condition: abstract, concrete, distraction), for the attentional disengagement index (Koster et al., 2005) measuring disengagement from negative and RNT-related words in relation to neutral words (see method section for disengagement index computation), with negative affect post-RNT induction as a covariate. As predicted, the analysis revealed a significant effect of word type (*F*_(1,54)_ = 4.29*, p* = 0.04, η_p_^2^ = 0.11), suggesting that participants are slower in disengaging from RNT-related words comparing to neutral words (mean attentional disengagement index = 3.05, *SD* = 32.11) and faster in disengaging from negative words relative to neutral words (mean disengagement index = −5.22, *SD* = 30.48). All the other effects were non-significant (*Fs* < 1).

To control for potential differences in dysphoria (Koster et al., 2005), we computed a mixed design ANCOVA with BDI-II score as a covariate. ANCOVA 2 (Word type: negative, RNT-related) × 3 (Experimental condition: abstract, concrete, distraction) computed for attentional disengagement index revealed a significant effect of Word type (*F*_(1,53)_ = 7.81*, p* = 0.01, η_p_^2^ = 0.13). Also the interaction effect between word type and covariate was significant (*F*_(1,54)_ = 4.19*, p* = 0.04, η_p_^2^ = 0.07). Participants seems to generally take longer to disengage from RNT-related words comparing to the negative ones, this effect is moderated by their dysphoria. All the other effects were non-significant.

**FIGURE 3:**
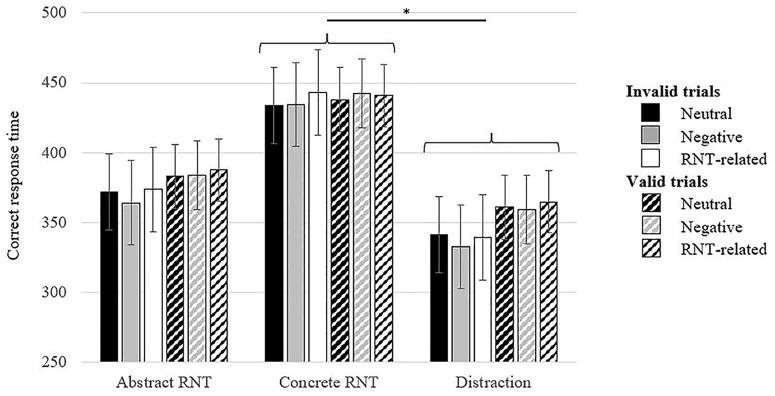
Mean correct response time in exogenous cueing task by RNT and distraction induction condition. *^∗^p* < 0.05.

Additionally, analyses were performed on the mean response times. A mixed design ANCOVA, 2 (Cue validity: valid, invalid) × 3 (Word type: neutral, negative, RNT-related) x 3 (Experimental condition: abstract, concrete, distraction) was computed on participants CRTs to exogenous cueing task, with negative affect post-RNT induction as covariate. A main effect of cue validity was observed, *F*_(1,55)_ = 4.50, *p* = 0.04, η_p_^2^ = 0.08. Participants detected quicker invalid cues comparing to the valid ones (*p* < 0.01), which suggests an inhibition of return effect. The results also revealed a significant main effect of Experimental condition, *F*_(2,55)_ = 3.61, *p* = 0.05, η^2^ = 0.10 (see Figure 3). *Post hoc* comparisons with Bonferroni correction suggested that participants in concrete RNT induction responded slower than participants from distraction group (*p* < 0.05). The results did not reveal any significant interactions.

### Moderating Effects of Trait RNT and Depressive Symptomatology on Attentional Disengagement

To explore how trait RNT and depressive symptomatology interact in their impact on attentional disengagement, we computed conditional process moderation models (Hayes, 2015) with trait-RNT and BDI-II score entered to the models as mean centered moderators. The experimental condition, as a categorical variable with 3 levels (abstract, concrete, distraction), was transformed into 2 dummy variables, following Hayes and Preacher’s (2013) guidelines. The first dummy variable created for abstract RNT (aRNT) induction was coded 1 for abstract RNT condition, and 0 for concrete RNT and distraction conditions. The second dummy variable created for concrete RNT (cRNT) induction was coded 1 for concrete RNT and 0 for abstract RNT and distraction conditions.

For testing a model with a predictor with k categories, it is necessary to run k-1 models (2 in our study). In both models one dummy variable was a predictor (X) and the remaining one was a covariate (C) (see Table 2), consequently we did not obtain one estimation of the effect, but an estimation for each category relative to the reference category in the dummy coding scheme (Hayes and Preacher, 2013).

**Table 2:** Conditional moderation process estimating difficulties in disengagement from negative words due to experimental condition, depressive symptomatology and trait RNT.

	Model with Dummy aRNT as X					Model with Dummy cRNT as X				
	Coefficient	*SE*	*p*	*LLCI*	*ULCI*	Coefficient	*SE*	*p*	*LLCI*	*ULCI*
Intercept	−0.79	7.40	0.91	−14.98	17.94	0.36	7.44	0.96	−13.99	14.08
Dummy aRNT (cRNT) (X)	2.84	11.61	0.81	−29.26	26.71	14.04	11.34	0.22	−5.10	35.77
Mini-CERTS AAT (M_1_’)	−11.79	7.68	0.13	−30.48	5.02	−6.27	6.60	0.35	−19.63	6.97
BDI-II (M_2_’)	20.49	7.54	<0.01	4.02	42.83	4.97	6.93	0.47	−9.40	21.33
Dummy x Mini-CERTS AAT (X M_1_)	2.46	11.84	0.84	−25.76	30.36	−22.18	13.24	0.10	−60.12	3.47
Dummy x BDI-II (X M_2_)	−22.40	12.52	0.08	−54.43	14.29	34.29	12.67	<0.01	10.36	64.16
Mini-CERTS AAT x BDI (M_1_ M_2_)	−6.77	4.83	0.16	−16.44	2.14	−10.83	5.71	0.06	−26.60	0.75
Dummy x AAT x BDI-II (X M_1_ M_2_)	−12.96	9.65	0.19	−36.04	11.89	−0.33	8.53	0.97	−17.51	16.82
Dummy cRNT (aRNT) (C)	9.14	9.04	0.32	−10.13	27.45	−2.95	9.54	0.76	−22.14	16.23
			*R*^2^ = 0.30, *MSE* = 766.38					*R*^2^ = 0.30, *MSE* = 760.54		
			*F*_(8,48)_ = 2.53, *p* = 0.022					*F*_(8,48)_ = 2.59, *p* = 0.019		

*aRNT, abstract RNT induction condition; cRNT, concrete RNT induction condition; AAT, Abstract thinking from Mini-CERTS; BDI-II, Beck Depression Inventory-II; LLCI, bootstrap lower level confidence interval; ULCI, bootstrap upper level confidence interval.*

First, attentional disengagement from negative stimuli was not affected by experimental induction (see Table 2). However, after including BDI-II and trait-RNT into the conditional process analysis, the results suggested that BDI-II score affected attentional disengagement from negative stimuli in a model with abstract RNT induction variable as a predictor (*p* < 0.01; see Table 2). BDI-II score also interacted with concrete RNT induction variable (*p* < 0.01). Thus, it seems that depressive symptomatology interferes with attentional disengagement. This interference is particularly apparent after concrete RNT induction, where dysphoric participants showed significantly higher index of attentional disengagement from negative stimuli comparing to the other conditions, suggesting that dysphoria is linked to impaired attentional disengagement from negative material (simple slopes for high BDI-II scores (+1SD) were significant: *p* < 0.05 for low abstract analytic thinking (AAT) and *p* = 0.05 for high AAT). This difference was not significant for participants with low BDI-II score (none of the simple slopes for low BDI-II scores (-1SD) were significant: *p* = 0.91 for low AAT and *p* = 0.14 for high AAT).

The conditional process moderation models predicting attentional disengagement from negative stimuli with trait RNT as moderator were non-significant, both, for abstract RNT (*p* = 0.058), and for concrete RNT induction (*p* = 0.15).

None of the models computed for attentional disengagement from RNT-related stimuli was significant: *R^2^* = 0.22, *MSE* = 898.45; *F*_(8,48)_ = 1.74, *p* = 0.11 for Dummy aRNT and *R^2^* = 0.13, *MSE* = 1007.68; *F*_(8,48)_ = 0.90, *p* = 0.52 for Dummy cRNT when BDI-II and AAT scores were included as moderators and *R^2^* = 0.21, *MSE* = 912.88; *F*_(8,48)_ = 1.61, *p* = 0.15 for Dummy aRNT and *R^2^* = 0.10, *MSE* = 1042.14; *F*_(8,48)_ = 0.67, *p* = 0.71 for Dummy cRNT when brooding and BDI-II scores were included as moderators.

In sum, there was no effect of abstract vs. concrete RNT induction on attentional disengagement when including moderating variables. Conditional moderation process models supported previous results that disengagement is affected by trait dysphoria (Koster et al., 2005). However, abstract vs. concrete RNT induction seems to affect the response time in exogenous cueing task, suggesting that participants using abstract RNT are faster to detect the stimuli independently of their valence.

## Discussion

The aim of the present study was to explore how experimental induction of abstract vs. concrete RNT after a situation typically conceptualized to activate rumination (i.e., involving actual-ideal self-discrepancy) impacts emotional reactivity (i.e., the changes in negative affect) and attentional disengagement. To the authors’ best knowledge this was the first study to experimentally test the link between two RNT processing modes and attentional disengagement. An additional aim was to explore whether the potential attentional impairment due to RNT is apparent only for negative stimuli (Koster et al., 2005), or is it specific for rumination-related stimuli as suggested by the attentional scope model of rumination (Whitmer and Gotlib, 2013).

According to the processing mode theory (Watkins, 2008), the adaptive character of rumination does not depend on the content of thoughts but is rather linked to the mode of information processing. The results of the present study seem to endorse this postulate for both, concrete and abstract RNT. We observed a distinctive impact of those processing modes on the change of pre-post induction negative affect (emotional reactivity). Participants using concrete RNT presented a lower emotional reactivity than those using abstract RNT. The results supported the theoretical predictions of processing mode theory and corroborate the results of previous studies (Watkins and Teasdale, 2004; Watkins and Moulds, 2005; Watkins et al., 2008).

However, the results concerning attentional disengagement are more complex and do not fully corroborate the theoretical predictions. It seems that concrete RNT– postulated as adaptive in a rumination situation – impairs participant’s attentional disengagement resulting in longer response time comparing to participants in control condition in both valid and invalid trials, independently from stimuli valence.

The most tempting would be to explain the difference in attentional performance by the fact that participants, after concrete RNT induction, are in a less negative affective state, as some studies suggested that positive mood can impair executive functioning (Phillips et al., 2002). However, in the present study the distinctive impact of abstract vs. concrete RNT on attentional disengagement cannot be imputed to differences in affect. Participants from distraction group, who did not differ from concrete group on affect, showed significantly shorter response times in the attentional disengagement task. Consequently, the deleterious effect of concrete processing cannot be attributed to the differences in affect between abstract and concrete conditions. Moreover, the statistical analyses suggest that affect did not influence the attentional indicators in our study.

### Emotional Reactivity vs. Attentional Processing

An important challenge is to explain why concrete RNT has beneficial effect on emotional reactivity, while, at the same time, and contrary to predictions, it impairs attentional disengagement. Even more challenging will be to determinate what are the consequences of this distinctive impact in the use of rumination focused clinical interventions like concreteness training (Watkins et al., 2009; Watkins, 2016). Training patients to use concrete thinking might certainly improve their short-term emotional reactivity, i.e., decrease their negative impact, but possibly impact also their attentional functioning. However, one might postulate that these differences in attentional processing are necessary to regulate negative affect and that it is rather the abstract RNT that causes an over-efficiency of disengagement from negative stimuli enhancing emotional avoidance. Nevertheless, in the present study, we have observed similar attentional results for abstract and distraction conditions, while the results were diverging in terms of emotional reactivity. Also, the lack of interaction between induction condition and type of stimuli might suggest that concrete RNT is in general linked to a less efficient attention control. The attentional impairment occurred for all types of stimuli.

On the one hand, the results are consistent with the control theory (Martin and Tesser, 1989) predicting that abstract processing should be associated with a greater self-control (increasing the focus on higher order goals and the resistance to immediate temptation). Although this pattern of results is relatively rare among experimental RNT research, it was previously observed in inhibition studies testing the link between inhibition and RNT (Altamirano et al., 2010; Zetsche and Joormann, 2011).

On the other hand, as postulated by the processing mode theory (Watkins, 2004), participants in concrete RNT condition are more focused on environmental details, their inner feelings at the moment, and consequently less focalized on the task being an external imposed goal (Watkins, 2011). They might experience some kind of immersion sensation that interferes with controlling and monitoring objectives. Concrete processing prevents from thinking of long-term consequences, and it is possible that it disturbs the cognitive efficiency. The lost in efficiency might also be due to the fact that concrete processing is an adaptive strategy of emotional regulation for ruminators, but this strategy requires additional cognitive resources and consequently participants cannot further allocate those resources to another task (here: exogenous cueing task). The impact on cognitive efficiency would be inversed for abstract processing. Previous studies showed that inducting abstract RNT results in a greater use of self-control in experimental tasks along with a greater perseveration comparing to concrete processing (Vallacher and Wegner, 1989; Fujita et al., 2006). A complementary explanation on the role of attentional control in ruminative process comes from the studies on thought suppression (Wegner et al., 1993; Wenzlaff and Luxton, 2003). In ruminators, the paradoxical effect of thoughts suppression is observed only under high cognitive load, when an attentional control dependent process of distracting one’s attention is inefficient and a more automatic and attentional control independent process of monitoring is operating (Wegner et al., 1993; Wenzlaff and Luxton, 2003).

The studies on ego-depletion provide additional arguments corroborating the idea that concrete processing (contrary to abstract one) results in lower emotional reactivity, but also in less efficiency in executive tasks. Schmeichel and Vohs (2009) suggest that abstract comparing to concrete processing can help to overcome self-control depletion and enhance the recruitment of attentional control resources. However, according to Webb and Sheeran (2003), it is the concrete processing that should result in recruiting cognitive resources. Probably the effect of concrete and abstract RNT on attentional control might be moderated by importance of the goal and its endogenous character.

It is important to underline, that our study corroborate previous findings suggesting that depressive symptoms are involved in attentional disengagement (Koster et al., 2005). An added value of the present study was to explore the interactions between RNT induction, trait RNT, and depressive symptomatology on attentional disengagement indicators. Although none of the models for attentional disengagement from RNT-related stimuli was significant, the results on attentional disengagement from negative stimuli suggest that disengagement might be impaired by dysphoria, corroborating previous results on attentional disengagement (Koster et al., 2005; Ferrari et al., 2016). Additionally, we observed that this effect was particularly apparent after concrete RNT induction (there was a tendency in the abstract RNT condition).

### Future Directions and Conclusion

In the present study, we aimed at differentiating the effect of attentional disengagement from general negative stimuli and disengagement from stimuli related to rumination (more relevant for participants) in order to test the predictions of attentional scope model of rumination (Whitmer and Gotlib, 2013). One of the limitations of the present study was the lack of standardization in the RNT-related stimuli. As we enhanced the ecological character of the stimuli by letting participants choose their own personal words, we were not able to standardize those stimuli valence. Participants seemed to choose words of different valence to describe their unresolved problem, with a mean valence score suggesting a neutral character of the words. It is important to further explore the idea of self-referent and RNT-related stimuli type, especially that Watkins (2016) suggested that abstract processing interferes with disengaging from an ongoing goal and not with attentional disengagement in general.

Additionally, the results of the present study support the hypothesis that, from a clinical perspective, some forms of cognitive control may be maladaptive (Messina et al., 2016). Participants using abstract RNT seems to present better attentional control, but, at the same time, their emotional reactivity is higher. Messina et al. (2016)’s suggestion that rumination might be linked with the ironic effect of exerting cognitive control might contribute to explain this effect and to open new therapeutic paths. However, before considering therapeutic implications and addressing the precise mechanism of maladaptive cognitive control in clinical settings, it is first necessary to evaluate the long-term interplay between different RNT types, cognitive resources and attentional control.

In the present study, we focused specifically on rumination — one of the emotional regulation strategies most related to psychological disorders — with the aim of distinguishing between abstract and concrete rumination according to processing mode theory (Watkins, 2008). This distinction seems crucial also from the clinical perspective of rumination-focused CBT, where concrete training (i.e., enhancing the use of concrete processing mode and reducing abstract processing mode) is one of the key elements (Watkins et al., 2009; Watkins, 2016). It seems interesting to further investigate the role of attentional control and its adaptive vs. maladaptive feature also beyond rumination and to test how it interplays with other emotional regulation strategies.

The present study was the first to explore the distinctive impact of abstract and concrete RNT on attentional disengagement. According to the results, the concrete RNT causes an attentional impairment for all types of stimuli (neutral, negatives and RNT-related). It is important to note that processing mode affected rather general attentional control and it is dysphoria that affected its particular component: attentional disengagement from negative stimuli. Dysphoria was the only significant predictor of the attentional disengagement from negative stimuli. However, concrete RNT enhanced a reduction of negative affect suggesting a lower emotional reactivity. Those results are particularly relevant in concreteness training perspective (Watkins et al., 2009; Watkins, 2016). It would be interesting to further explore whether an impairment in attention is a consequence of the cognitive cost of an adaptive emotional regulation in ruminators.

## Ethics Statement

The study was run with University of Lille Ethic Committee approval (decision number: 2014-3-S23) and in accordance with the recommendations of the American Psychological Association and the Declaration of Helsinki. Participants signed a consent form prior to their participation, the procedure ended by a debriefing. The experimenter explained the aim of the study and answered all questions.

## Author Contributions

MK designed the research, collected the data, did statistic analysis, wrote and revised the manuscript. IK did statistic analysis and revised the manuscript. CD designed the research, did statistic analysis and revised the manuscript.

## Conflict of Interest Statement

The authors declare that the research was conducted in the absence of any commercial or financial relationships that could be construed as a potential conflict of interest.
