# Supplementary material for: Concrete vs. Abstract Processing in Repetitive Negative Thinking: Distinct Functional Effects on Emotional Reactivity and Attentional Control
Source: Front Psychol. 2019 Jun 18;10:1372. doi: 10.3389/fpsyg.2019.01372 (PMC6591261; doi:10.3389/fpsyg.2019.01372)
Supplement: Supplementary file 1 [file Table_1.DOCX]

**Appendix 1**

1. Abstract RNT induction

*Analyze the causes, the consequences and signification of*

the physical sensations in your body

*Analyze the causes, the consequences and signification of*

the degree of clarity in your thinking right now

*Analyze the causes, the consequences and signification of*

the way you react

*Analyze the causes, the consequences and signification of*

the amount of tension in your muscles

*Analyze the causes, the consequences and signification of*

your present feelings of fatigue or energy

*Analyze the causes, the consequences and signification of*

how hopeful or hopeless you are feeling

*Analyze the causes, the consequences and signification of*

your physical sensations

*Analyze the causes, the consequences and signification of*

the level of motivation you feel right now

*Analyze the causes, the consequences and signification of*

the way you feel

*Analyze the causes, the consequences and signification of*

how passive or active you feel

*Analyze the causes, the consequences and signification of*

the degree of control you feel right now

*Analyze the causes, the consequences and signification of*

how quick or slow your thinking is right now

1. Concrete RNT induction

*Focus your attention on*

the physical sensations in your body

*Focus your attention on*

the degree of clarity in your thinking right now

*Focus your attention on*

the way you react

*Focus your attention on*

the amount of tension in your muscles

*Focus your attention on*

your present feelings of fatigue or energy

*Focus your attention on*

how hopeful or hopeless you are feeling

*Focus your attention on*

your physical sensations

*Focus your attention on*

the level of motivation you feel right now

*Focus your attention on*

the way you feel

*Focus your attention on*

how passive or active you feel

*Focus your attention on*

the degree of control you feel right now

*Focus your attention on*

how quick or slow your thinking is right now

1. Distraction induction

*Imagine*

a boat slowly crossing an ocean

*Imagine*

the layout of a typical classroom

*Imagine*

the shape of a large black umbrella

*Imagine*

the movement of an electric fan

*Imagine*

the shape of African continent

*Imagine*

raindrops sliding down a window pane

*Imagine*

the shape of Eiffel Tower

*Imagine*

a full moon on a clear night

*Imagine*

clouds forming in the sky

*Imagine*

the layout of a local shopping centre

*Imagine*

a plane flying overhead

*Imagine*

a train stopping at a train station
